# Development of a Species-Specific SCAR-PCR Assay for Direct Detection of Sugar Beet Cyst Nematode (*Heterodera schachtii*) from Infected Roots and Soil Samples

**DOI:** 10.3390/life11121358

**Published:** 2021-12-07

**Authors:** Chen Jiang, Yingdong Zhang, Ke Yao, Sulaiman Abdulsalam, Guangkuo Li, Haifeng Gao, Kemei Li, Wenkun Huang, Lingan Kong, Deliang Peng, Huan Peng

**Affiliations:** 1 State Key Laboratory for Biology of Plant Diseases and Insect Pests, Institute of Plant Protection, Chinese Academy of Agricultural Sciences, Beijing 100193, China; 18684759602@163.com (C.J.); zhangyingdong26@163.com (Y.Z.); 17794539779@163.com (K.Y.); asulaiman@abu.edu.ng (S.A.); wkhuang2002@163.com (W.H.); konglingan@caas.cn (L.K.); 2 College of Agriculture and Biotechnology, Zhejiang University, Hangzhou 310058, China; 3 Key Laboratory of Integrated Pest Management on Crop in Northwestern Oasis, Ministry of Agriculture, Institute of Plant Protection, Xinjiang Academy of Agricultural Sciences, Urumqi 830091, China; lgk0808@163.com (G.L.); ghf20044666@163.com (H.G.); 4 College of Agriculture, Xinjiang Agricultural University, Urumqi 830091, China; likemei@xjau.edu.cn

**Keywords:** sugar beet cyst nematode, *Heterodera schachtii*, RAPD marker, SCAR-PCR

## Abstract

Sugar beet cyst nematode (SBCN, *Heterodera schachtii*) is an important nematode that causes significant yield losses of 25–50% or more in most areas of sugar beet production worldwide. Rapid and accurate identification of this species is essential to support decisions on pest management. However, the difference between *H. schachtii* and other *Heterodera* spp. based on morphology is a challenging task. In the present study, a SCAR-PCR assay was developed to identify and differentiate *H. schachtii* in infected root and soil samples. *H. schachtii*-species-specific SCAR-PCR primers OPA06-HsF and OPA06-HsR were designed from the randomly amplified polymorphic DNA (RAPD) marker amplified with random primer OPA06. The developed primers specifically amplify a 922-bp fragment from the target populations but did not amplify DNA from non-target cyst nematodes including *Heterodera*, *Globodera*, *Cactodera*, and other related species tested in this study. The sensitivity detection indicated that 5 × 10^−4^ of a single cyst, 1/320 of a single second-stage juvenile (J2), or 10 pg of genomic DNA could be detected. The assay accurately identifies the different stages of *H. schachtii* in sugar beet and oilseed rape roots as well as a single J2 in 10 g of soil. Finally, the SCAR-PCR assay detected *H. schachtii* in seven samples out of the fifteen field samples. The assay will not only be useful for differentiating *H. schachtii* from mixed populations of *Heterodera* spp. but also for effective detection of the species directly from infested samples. The assay also requires no expertise in the taxonomy and morphology of the species but serves to improve the diagnosis of *H. schachtii* in infested fields.

## 1. Introduction

Sugar beet cyst nematode (SBCN, *Heterodera schachtii*, Schmidt, 1871) is an economically important plant-parasitic nematode that limits the production of sugar beet across the world [1,2]. It was first identified in Germany in 1859 by Schachti as a plant pathogen when it caused yield reduction in sugar beet cultivation. Today SBCN is distributed in more than 50 countries and was recognized and listed as a quarantine pest by more than 20 countries of the world, including China, to restrict its spread [3,4]. The nematode has a wide host range, infecting over 218 plant species belonging to 95 genera and 23 different plant families [5,6]. It was also reported to cause yield losses of 25–50% or more in sugar beet and other crops grown [7].

SBCN belongs to the *H. schachtii* sensu stricto group, which also includes *H. glycines*, *H. betae*, *H. ciceri*, *H. daverti*, *H. medicaginis,* and *H. trifolii* [8]. Morphological differences between *H. schachtii* and its closely related species can be highly challenging. For example, *H. trifolii* exhibit similar body characteristics and damage symptoms with *H. schachtii* particularly at the field level. However, there are some small morphological and morphometric differences that are useful to differentiate these species in this group at the laboratory level [7]. Therefore, the species identification process is time consuming, laborious, and requires a high level of expertise. In the last few decades, a series of DNA-based molecular detection assays have been reported for the identification and discrimination of *Heterodera* species, including SBCN. Genetic diversity of SBCN populations was carried out using Random Amplified Polymorphic DNA (RAPD) and Amplified Fragment Length Polymorphism (AFLP) techniques [9,10], Polymerase chain reaction internal-transcribed spacer-restriction fragment length polymorphism (PCR-ITS-RFLP) using MvaI restriction enzyme to distinguish *H. schachtii* from other cyst nematodes [11], and PCR by a species-specific primer (SHF6) in combination with the universal primer (rDNA2) that amplifies a DNA fragment of 255 bp [12], and this developed primer (SHF6) was modified and used for qPCR-based detection of *H. schachtii* with SYBR Green dye [13]. However, Toumi et al. [14] reported that the primer (SHF6) was unable to detect some *H*. *schachtii* populations, as this polymorphism is not present in all *H. schachtii* populations. Gamel et al. [15], also succeeded in developing a multiplex species-specific qPCR assay based on microsatellite loci that simultaneously detect and identify *H. schachtii*, *Globodera pallida*, and *G. rostochiensis* in a mixed population; the limit of detection was five juveniles for *H. schachtii*. As we all know, real-time PCR is an advancement over conventional PCR; however, this assay is still not suitable for poorly equipped laboratories.

The ITS region has been widely used in the design of species-specific PCR primers and has been proven useful in the development of diagnostic tests for the detection of these nematode species [12,16,17]. RAPD fingerprints as earlier stated provide a good tool for studying genetic variation at the species level [18,19,20]. The method has been employed to distinguish between *H. cruciferae* and *H. schachtii* [9]. RAPD yielded reliable and repeatable patterns that separate species or populations of SBCN when the PCR conditions were kept constant. By comparing these patterns, unique fragments can be identified and used to design species-specific primers. The primers can be used to generate the sequence characterized amplified regions (SCAR) for SBCN detection. SCAR assays have also been applied for the detection of many important cyst nematodes, such as *G. rostochiensis* and *G. pallida* [21], *H. glycines* [22,23], and *H. filipjevi* [24]. However, the use of SCAR-PCR assay has not yet been reported for BSCN detection.

SBCN is considered one of the most important quarantine nematodes in China as well as in the world. The nematode was first reported in Guangdong Province of China in the 1990s [25]; however, this record has yet to be substantiated. Recently, this species has been reported in Xinyuan county, Xinjiang Uygur autonomous region of China based on the morphological and molecular characterization [26]. It is difficult to detect, particularly in early infected host roots; therefore, there is an urgent need to develop a rapid and accurate diagnostic approach for direct detection of the nematode in both plant root and soil samples. This study aimed to develop a sensitive and specific SCAR-PCR assay based on RAPD fragments for the direct detection of *H. schachtii* from infected plant root and soil samples.

## 2. Materials and Methods

### 2.1. Nematode Populations

Nine populations of *H. schachtii* from four countries, 12 other cyst-forming nematode species, and five other related nematode species were used in this study (Table 1). The species identities were based on morphology and verified by ribosomal DNA sequencing on the ITS region. The second-stage juveniles (J2s) of *H. schachtii* were hatched in a 3 mM ZnCl_2_ solution at 25 °C.

### 2.2. DNA Extraction

A single cyst, female or J2, was hand-picked into 0.2-mL PCR tubes containing a total of 20 µL lysis buffer (including 7 µL 10 × PCR buffer (100 mM Tris-HCl (pH8.9), 500 mM KCl, and 15 mM MgCl_2_) (Takara-Bio, Shiga, Japan), 3 µL Proteinase K (600 µg/mL), and 10 µL distilled water) separately; crude DNA extract was performed from a single nematode as described by Ou et al. [23]. The pure genome DNA of *H. schachtii* was isolated from the mass of J2s by the phenol-chloroform method, and the pure genome DNA was quantified using the NanoDrop ND-2000 Spectrophotometer (Thermo Scientific, USA). The genomic DNA of nematode in 0.1 g of plant root was isolated using Universal Genomic DNA Extraction Kit (Takara-Bio, Shiga, Japan), and the Power Soil^®^ DNA Isolation Kit (MoBio Laboratories Inc. Qiagen, Germantown, MD, USA) was used to isolate DNA from soil samples according to the manufacturer’s instructions. The genome DNA was stored at −20 °C.

### 2.3. RAPD-PCR-Analysis

Fifteen different random primers (Table 2) for SBCN assays were selected based on previous reports [9,23,41]. The PCR amplification reaction contained 5 µL 10 × PCR buffer, 4 µL 2.5 mM dNTPs, 1 U ExTaq DNA polymerase (Takara-Bio, Shiga, Japan), 3 µL 10 µM each primer, 1 µL template DNA, and double-distilled water (ddH_2_O) to a total volume of 50 µL. The PCR was performed in an S1000^TM^ thermal cycler (Bio-Rad Laboratory, Inc. Hercules, CA, USA) under the following conditions: 5 min at 95 °C, 10 cycles of 30 s at 95 °C, 40 s at 45 °C, 1 min at 72 °C, and 20 cycles of 30 s at 95 °C, 40 s at 55 °C, 1 min at 72 °C, followed by 10 min at 72 °C, and stored at 4 °C. ddH_2_O was used as a negative control to avoid misinterpretations of the RAPD patterns that may be caused by manual manipulation. A total of 10 µL of the PCR products were separated on a 2% agarose gel, visualized by Gelred gel staining (GelStain, TransGen Biotech Beijing, China), and photographed under UV light. All reactions were repeated twice to confirm the results of the reaction with our target bands.

### 2.4. SCAR Primer Design

Based on the results of initial RAPD-PCR-analysis, a prominent candidate band amplified by primer OPA 06 that clearly distinguished the SBCN from the other 3 tested cyst nematode species, was selected for the development of the SCAR marker. The band was purified and cloned into the pMD19-T vector (Takara-Bio, Shiga, Japan). Sequencing was performed by Shanghai Sangon Co., Ltd., and the sequences were analyzed using BLAST on the NCBI website [43]. The sequences alignment analysis was performed with MEGA X [44]. A set of specific SCAR primers OPA6-HsF and OPA6-HsR was designed using Primer 6.0 and tested on NCBI with Primer-BLAST program (http://www.ncbi.nlm.nih.gov/tools/primer-blast, accessed on 14 August 2021).

### 2.5. Specificity Test of SCAR Primer

Nine populations of *H. schachtii* collected from Germany, Turkey, Belgium, China, and another 17 plant nematode species were used for SCAR primers specificity analysis. The PCR amplification reaction was carried out as follows: 50-µL reaction contained 5 µL 10 × PCR buffer, 4 µL 2.5 mM dNTPs, 1 U ExTaq DNA polymerase (Takara-Bio, Shiga, Japan), 0.6 µL of 10 µM each pair of SCAR primers (OPA06-HsF/OPA06-HsR), 0.6 µL of 10 µM each pair of universal primer (D2A/D3B), 1 µL template DNA, and 37.6 µL of ddH_2_O. The PCR was performed under the following conditions: The thermocycler was programmed for 5 min at 95 °C, 35 cycles of 30 s at 95 °C, 40 s at 56 °C, 1 min at 72 °C, followed by 10 min at 72 °C, and stored at 4 °C. PCR products were analyzed by electrophoresis previously described. The experiment was repeated three times to confirm the result.

To evaluate the specificity of SCAR-PCR from mixed populations, first, a single cyst of SBCN (HS2) was mixed with other cyst nematode species *Heterdera* and *Globodera* genus (Groups 1 and 2). The RAPD sequences blast result shows that the sequences have homology with several *Meloidogyne* species. Thus, the treatment group comprises a mixture of root-knot nematodes including a single female of *M. enterolobii*, *M. incognita*, *M. hapla*, and *M. graminicola* together with a cyst of SBCN (Group 3). Finally, the *D. ditylenchus* species was always found in the sugar beet planting area in China; therefore, this species was also included (Group 4). The *H. schachtii* was absent from those groups that were used as the control (Groups 5, 6, 7. and 8). The specific treatments were listed in Table 3.

### 2.6. SCAR Primer Sensitivity Test

To determine the sensitivity of the specific primers, DNA of a single cyst and a single juvenile were diluted in a certain proportion respectively, and ten-fold serial dilution of *H. schachtii* genome DNA (starting at 1000 ng/µL) were prepared with dilution buffer (Takara-Bio, Shiga, Japan). DNA from a single cyst and a single J2 were serially diluted in 10-fold and 2-fold increments in sterile water (starting at 1 µL DNA from a single cyst or J2, that is, 1/20 a single cyst or J2), respectively. All different dilutions of DNA were used as DNA templates for SCAR-PCR reaction with OPA06-HsF and OPA06-HsR primers separately. The experiment was repeated three times for each sample.

### 2.7. Duplex PCR Amplification

In this present study, the universal primers D2A and D3B [16] were used to amplify a 780-bp fragment of the 28S rDNA gene to test the quality of the DNA template while amplifying specific fragments of the target nematode species from the non-target species using OPA06-HsF/OPA06-HsR primers. The PCR amplification reaction was performed as described above for the species-specific SCAR-PCR assay.

### 2.8. Direct Detection of H. schachtii in Artificially Inoculated Plant Roots and Soil

Sugar beet (cv. SD12830) and oilseed rape (cv. Deyou NO. 6), both of which are host plants of BCN, were planted in 250-mL plastic containers and cultured in the greenhouse. Two weeks later, 500 J2s were inoculated per plant, and the inoculated roots samples were collected separately at 3 days after inoculation (DAI), 9 DAI, and 15 DAI, respectively. For nematode assay, each sample was divided into two equal parts; one part (0.1 g) was used for counting the presence of nematodes in the inoculated root samples after staining with acid fuchsin. The remaining parts were stored at −80 °C. A total of 0.1 g of the root was selected for DNA extraction, and three biological replicates were used for each treatment. Furthermore, 1, 5, 10, 25, and 50 J2s of sugar beet cyst nematode were hand-picked into 10 g of autoclaved sand soil. The genomic DNA isolated from the artificially inoculated soil samples were all used as templates to test the SCAR-PCR assay. The PCR amplification was performed using highly active KOD FX DNA polymerase (TOYOBO, Osaka, Japan) containing 50 µL PCR reaction mixture: 2 × KOD FX Buffer 25 µL, 2 mM dNTPs 10 µL, 10 µM each primer 1.5 µL, template DNA 2.5 µL, 1 U KOD FX DNA polymerase, and 11 µL distilled water. The thermocycler was run under the following conditions: 94 °C for 2 min, 40 cycles of 10 s at 98 °C, 40 s at 60 °C, 30 s at 68 °C, followed by 10 min at 68 °C, and stored at 4 °C. The healthy root and autoclaved soil samples were used as negative controls. Additionally, the potential ability of the published specific primers SHF6 and rDNA2 [12] for direct detection of *H. schachii* from the plant or soil DNA was tested with the KOD FX DNA polymerase. The experiments were repeated three times.

### 2.9. Direct Detection from Naturally Infected Roots and Soil Samples

To evaluate the practical application of the SCAR primers, ten field samples, including sugar beetroots and rhizosphere soil, were collected from the counties surrounding Xinyuan County, Xinjiang province in June 2020. In addition, another five samples were also collected from Ermin County of Xinjiang and Inner Mongolia province in July 2021 (Table 4). These are the main sugar-beet-growing areas in China. Ten sugar beet roots together with approximately 500 g of rhizosphere soil samples were carefully collected from each sugar beet field surveyed and kept in the refrigerator at 4 °C for nematode assay. Ten grams (10 g) of soil together with one hundred milligrams (100 mg) of sugar beet roots were randomly selected from each well-mixed sample for DNA extraction. Genome DNA extraction and SCAR-PCR detection with KOD FX DNA polymerase were performed as described above. As a comparison, numbers of SBCN were extracted and counted from each root (0.1 g) sample using the acid fuchsin staining method and cysts from 100 g soil sample using the floatation method. A single cyst was randomly picked from each sample, and the DNA was isolated and used as the templates for PCR amplification using previously developed specific-*H. schachtii* primers (SHF6 and rDNA2, Table 1) and rDNA-ITS universal primers (TW81 and AB28, Table 1) to confirm the results. The genomic DNA of SBCN and ddH_2_O was used as a positive and negative control, respectively. Each sample was repeated three times.

## 3. Results

### 3.1. RAPD-PCR and Sequences Analysis

**Figure 1:**
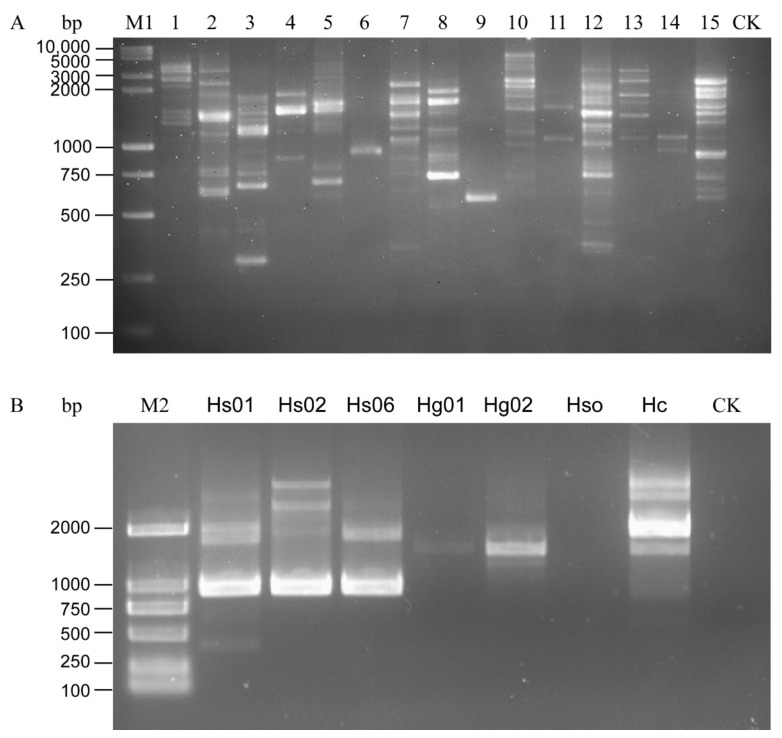
Amplification pattern of RAPD primers. (**A**) PCR results of *Heterodra schachtii* using 15 RAPD primers. (**B**) RAPD pattern of *H. schachtii* and *H. glycines* using Primer OPA06. 1–15: OPA-01, OPA-02, OPA-3, OPA-04, OPA-05, OPA-06, OPA-09, OPA-13, OPA-18, OPB-15, OPC-06, OPD-13, OPG-06, OPG-08, and OPK-16. CK, negative control (ddH_2_O instead of DNA). M1, DL10,000 + 2000 DNA Ladder; M2, D2000 DNA Ladder.

All the *H. schachtii* populations were used to test the 15 primers. The following primers, OPA01, OPA02, OPA03, OPA05, OPA09, OPA13, OPB15, OPD13, and OPK16, produced multiple RAPD amplification bands and the amplification results were unstable in the different populations, as shown in the repeated experiments. OPA06 can stably amplify 1000-bp bands from DNA samples of the *H. schachti* population, thus clearly distinguishing the SBCN from other tested cyst nematode species (Figure 1). The bands were purified, sequenced, and submitted to Genbank (Accession number MW854319). The Blastn results indicated that this sequence was similar to *H. schachtii* genomic sequences (GenBank accession No. JAHGVF010000211.1) and *H. glycines* genomic DNA sequences (VAPQ01000257.1) with 99.24% and 86.35% identity, respectively. The sequences also have homology (identity <73%) to genomic sequences of *G. pallida* (CBXT010012390.1), *G. ellingtonae* (MEIZ01000021.1), *G. rostochiensis* (JAEVLO010000036.1), *M. incognita* (RCFL01002996.1), *M. arenaria* (QEUI01000363.1), *M. javanica* (RCFK01013271.1), *M. floridensis* (RCFN01001171.1), and *M. enterolobii* (CAJEWN010003051.1) with low query cover (<29%). The sequences alignment is shown in Figure S1.

### 3.2. Primers Design and Species-Specific Test

**Figure 2:**
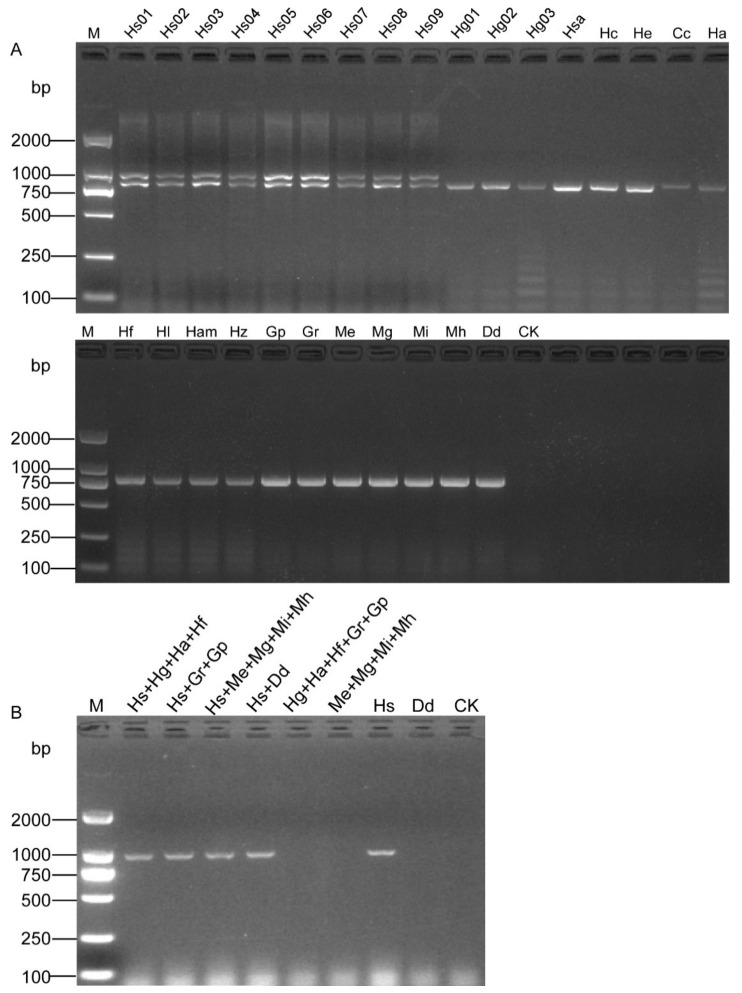
Specificity assessment of the SCAR marker for the detection of SBCN. (**A**) Duplex PCR results of SCAR and D2A/D3B primers. Two bands of 922 bp and 780 bp were observed from nine populations of *H. schachtii* (Hs01–Hs09), and a single fragment of 780 bp was obtained from other nematode samples (Hg01, Hg02, Hg03, Hso, Hc, He, Cc Ha, Hf, Hl, Ha, Hz, Gr, Gp Mi, Me, Mh, Mg, and Dd). (**B**) PCR products from mixed populations using the SCAR-PCR. CK, negative control (ddH_2_O instead of DNA). M, D2000 DNA Ladder.

A pair of specific SCAR primers OPA06-HsF/OPA06-HsR (Figure 1 and Table 1) was designed based on the sequence dissimilarity to *H. schachtii* and other related nematode species (Figure S1). The primer-BLAST result showed that the primer pair (OPA06-HsF and OPA06-HsR, Table 2) was specific to the OPA06 fragment, as no other targets were found in the Nr database. All the DNA samples were tested, and a specific band of 922 bp was only obtained from *H. schachtii* populations. The duplex PCR with samples containing specimens of the *H. schachtii* populations yielded two distinct fragments of 922 bp and 780 bp, respectively. In contrast, a single fragment of 780 bp was also obtained from other nematode samples tested (Figure 2A). The PCR amplification with DNA templates from mixed populations showed that the positive bands were observed from the samples containing SBCN (Figure 2B).

### 3.3. Sensitivity Test

**Figure 3:**
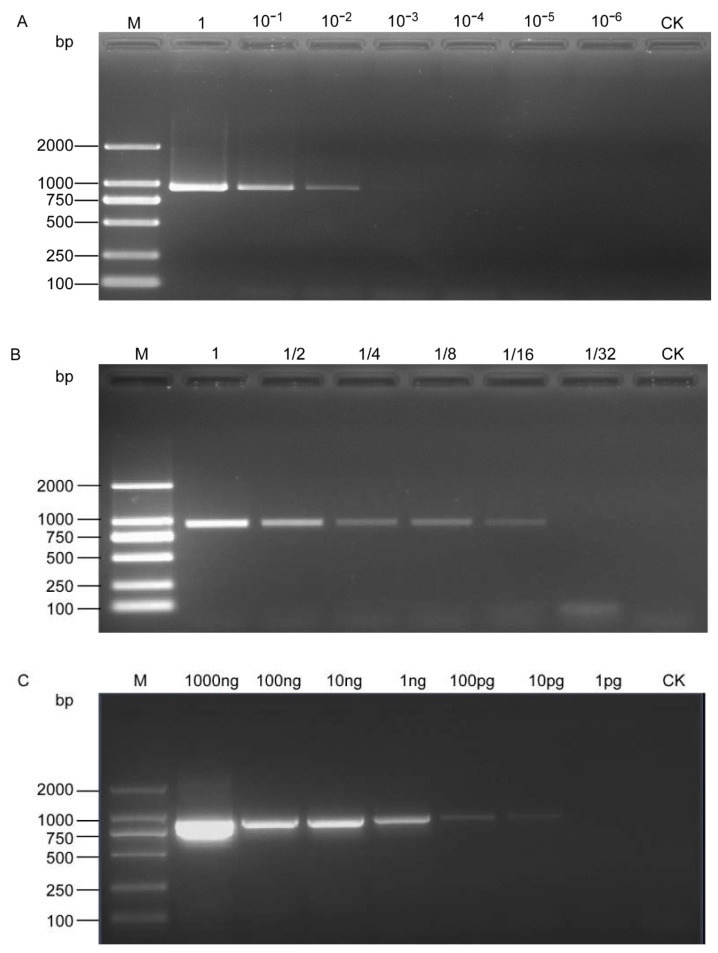
Sensitivity assessment of the SCAR-PCR method for the detection of *H. schachtii*. (**A**) Performed with serial dilution of DNA from a single cyst; (**B**) performed with serial dilution of DNA from a single second-stage juvenile; (**C**) sensitivity test with serial dilution of a genomic DNA template. M, D2000 DNA Ladder; CK, negative control (ddH_2_O instead of DNA).

The PCR results showed that the SCAR-PCR assay could detect *H. schachtii* as low as 10^−2^ μL DNA from a single cyst and 1/16 μL DNA from a single J2, that is, 5 × 10^−4^ of a cyst and 1/320 of a J2 in the reaction mixture (Figure 3A,B). Quantitative DNA templates were also used and the results showed that visible bands could be amplified at a DNA concentration of even as low as 10 pg/µL (Figure 3C).

### 3.4. Direct Detection of H. schachtii in Inoculated Plant Roots and Soil

**Figure 4:**
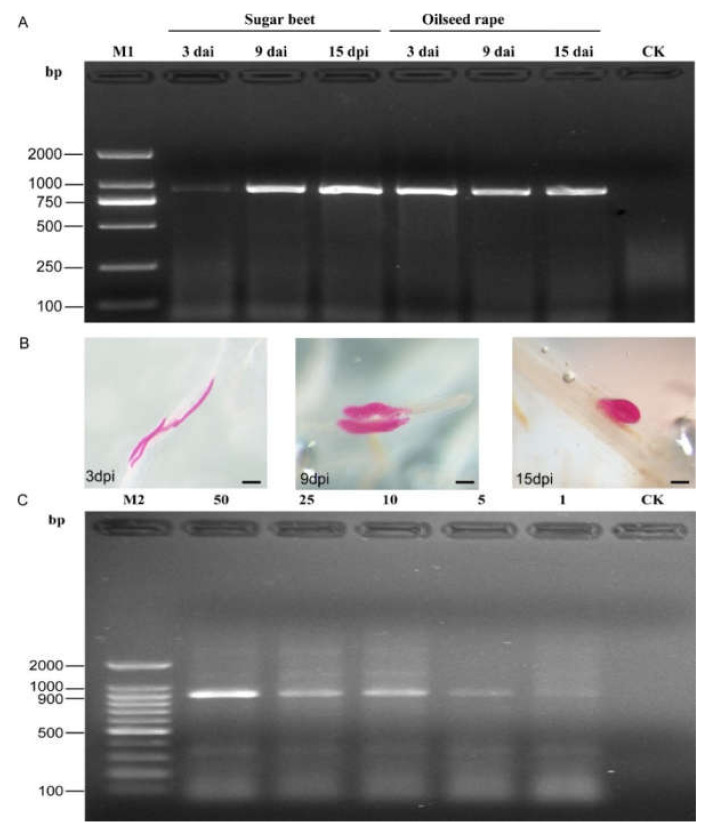
PCR products from inoculated roots and soil samples using the SCAR-PCR method. (**A**) PCR performed at 3 days, 9 days, and 15 days after inoculation (DAI) with *H. schachtii* using SCAR marker; Non-inoculated roots as the control; (**B**) *H. schachtii* developing stages in sugar beet roots at 3, 9, and 15 DAI (stained), bar = 100 μm; (**C**) detection of *H. schachtii* from DNA extracts from 50, 25, 10, 5, and 1 J2s in 10 g of soil; autoclaved soil as the control (CK). M1, D2000 DNA ladder; M2, 100-bp DNA ladder.

The SCAR-PCR test results indicated that positive bands of 922 bp were observed in all root samples inoculated by SBCN (Figure 4A). Moreover, numerous *H. schachtii* were observed in the remaining part of the root samples after staining with acid fuchsin (Figure 4B). Additionally, the SCAR-PCR assay can accurately detect one J2 per 10 g of soil sample. Negative results were obtained from the non-inoculated root and autoclaved soil sample (Figure 4C). In addition, the potentiality of the published species-specific primer SHF6/rDNA2 was not performed using genomic DNA extracted from plant roots and soil samples (Figure S2).

### 3.5. Direct Detection of Naturally Infected Root and Soil Samples

**Figure 5:**
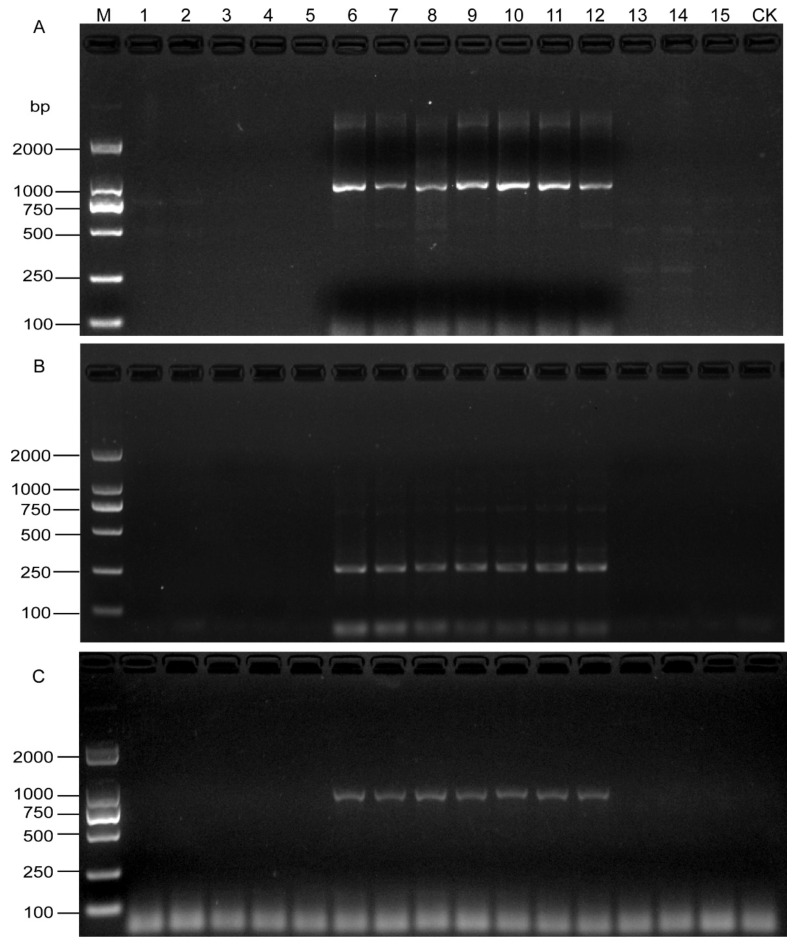
PCR products from naturally infested field root and soil samples using the SCAR-PCR method. (**A**) Detection of *H. schachtii* from root using SCAR primers. (**B**) Detection of *H. schachtii* from cyst using SHF6 and rDNA2 primers. (**C**) Direct detection of *H. schachtii* from field soil samples using SCAR primers. M, D2000 DNA Ladder; CK, Negative control.

A 922-bp band was observed in seven root samples from Xinyuan and Ermin Counties of Xinjiang Province. No bands were obtained from the other eight field samples and negative control (Figure 5A). Moreover, the detection results of soil samples from the same locations were consistent with those of roots (Figure 5B), which showed that the SCAR primers could effectively detect SBCN from the field samples. The results were confirmed by the developed specific primers with the DNA template from a single cyst (Figure 5C and Table 4).

## 4. Discussion

SBCN infects a wide spectrum of hosts, posing a severe economic threat to sugar beet production worldwide [6,7,45]. This species has been recognized as a quarantine nematode in China and some other countries; unfortunately, it was recently discovered in Xinyuan county Xinjiang province, China [26]. For management decisions, an effective and reliable diagnostic assay to detect the presence of SBCN in root and soil is important. In this present study, based on the amplification sequence of the RAPD primer OPA06, a set of species-specific SCAR primers for SBCN detection was developed in the current study. The assay was highly specific and able to distinguish *H. schachtii* from other morphologically similar *Heterodera* spp. and other nematodes species. Furthermore, the assay was validated using artificially and naturally infested roots and soils with varying *H. schachtii* population densities.

To estimate the specificity of the SCAR-PCR assay developed in this study, the blast search results indicated that the sequence was similar to *H. glycines* with 86.35% identity. Previous reports have shown that *H. schachtii* and *H. glycine* diverged from a common ancestor and to be sibling species within the same taxonomic subgroup (*schachtii*). Thus, it was a great challenge to differentiate *H. schachtii* and *H. glycine* due to their similarity and close relationship [29]. Conversely, a specific band was also obtained in only nine populations of *H. schachtii* isolated from four different countries (Germany, Turkey, Belgium, and China) while the negative results were obtained from 19 isolates belonging to 10 closely related cyst nematodes and other five nematode species. The results show that our developed primers of *H. schachtii* have been proven to be exceptionally specific when tested with template DNA of the aforementioned isolates. Even the primers developed in this study were selected in the specific genomic sequences where there exist differences between *H. schachtii* and other cyst and related species (Figure 2); in the future, we recommend that more populations of *H. schachtii* and other closely related cyst nematode species should be tested to avoid the risk of misidentification.

This sensitivity detection of our developed assay was higher than previous reports for SBCN identification. Amiri et al. [12] could detect 0.6 ng genomic DNA or DNA of 1/1000 cyst of *H. schachtii* using a species-specific primer set designed from ITS regions. Furthermore, Madani et al. [13] also report that the sensitivity of their real-time PCR diagnostic tool for *H. schachtii* detection was lower than 100% for samples containing fewer than five J2; however, a single J2 and three J2 were detected in only 20 and 60% of the samples, respectively, the limit of detection was consistent with the results of Gamel et al. [15]. In contrast, the SCAR-PCR assays developed in the present study were sensitive enough to detect as low as 1/320 of a J2 of *H. schachtii*, even if the quantitative PCR is an advancement of the conventional PCR method.

To evaluate the practical application of the SCAR primers for direct detection of SBCN from the infested root and soil samples, the result shows that the nematodes were successfully amplified from two infected plant roots at 3, 9, and 15 DAI. Microscopic observations also show that *H. schachtii* becomes parasitic during the following growth stages: J2 at third stage (J3) and adult females at 3, 9, and 15 DAI, respectively. These diagnostic observations reported in this study indicate that SBCN could be detected at different growth stages in sugar beet and oilseed rape roots. The same scenario was also reported for the detection of cereal cyst nematode *H. filipjevi* [24] and false root-knot nematode *Nacobbus* species [46]. Additionally, this SCAR assay can accurately identify one J2 of *H. schachtii* in 10 g of the soil sample, equivalent to 20 SBCN juveniles/200 g of soil. It is consistent with the previous report for detection of *H. glycines* using SCAR-PCR assay [15] but higher than those previously reported. Ophel-Keller et al. [47] detected fewer than one egg of *H. avenae* per gram of soil by real-time PCR. Peng et al. [24] were also able to detect single juveniles of *H. filipjevi* per 0.5 g of soil equivalent to 400 juveniles/200 g of soil. However, the potentiality of the previously published species-specific primer SHF6/rDNA2 for *H. schachii* detection was not performed in this study using genomic DNA extracted from plant root and soil samples either with Taq DNA polymerase or KOD FX DNA polymerase (Figure S2). This implies that, if the reported primers were to be used, more time will be spent to isolate the target nematode from the soil samples. Interestingly, the SCAR-PCR assay developed in this study was able to overcome such limitations and thus shows high detection sensitivity to SBCN. Our results indicated that the SCAR method can serve as a detection tool to diagnose early SBCN infection in the plant root and field soils, where there is absolutely no visual symptom of infection.

The SCAR-PCR assay was also validated by testing its SBCN detection in 15 naturally infested sugar beet root and soil samples. The positive results were observed in seven beet root samples from Xinyuan and Ermin Counties of Xinjiang Province but absent in other eight field samples from Huocheng and Qapqal Counties of Xinjiang and three counties of Inner Mongolia. The results indicated that the presence of the SBCN has now been confirmed in Xinyuan and Ermin County of Xinjiang Province; however, its distribution in China is limited based on the present report [26]. *H. schachtii* is an economically important plant-parasitic nematode that limits sugar beet production worldwide; thus, it has now been recognized and listed as a quarantine pest in China. Furthermore, Xinjiang province is one of the major sugar beet cultivation areas in China; therefore, strict control measures should be taken to prevent further spread of the nematode to other beet growing areas of the country.

## 5. Conclusions

This study presents the first protocol using a species-specific SCAR-PCR assay to detect *H. schachtii* directly from artificially and naturally infested root and soil samples of sugar beet plants. The SCAR-PCR assay will also be useful for discriminating *H. schachtii* from mixed populations of other cysts and non-cysts nematode species. It is simple, rapid, reliable, and accurate irrespective of the life stage and abundance of the nematode and has lower requirements for experimental conditions. Therefore, the assay eliminates the time-consuming stages of traditional nematode extraction, microscopic identification, and counting and requires no prior knowledge of nematode taxonomy or morphology. It can be used not just in research but also in diagnostic laboratories, infested fields, and pest management extension services in China.

## Figures and Tables

**Table 1:** Plant nematode populations used.

Species	Species Code	Population Origin	Host	References
*Heterodera schachtii*	Hs1	Germany	Sugar beet	[27]
	Hs2	Xinyuan county Xinjiang, China		This study
	Hs3	Xinyuan county Xinjiang, China		
	Hs4	Xinyuan county Xinjiang, China		
	Hs5	Xinyuan county Xinjiang, China		
	Hs6	Belgium ^a^		[13]
	Hs7	Bozova, Anliurfa Province, Turkey		[28]
	Hs8	Karakprü, Anliurfa Province, Turkey		
	Hs9	Siverek, Anliurfa Province, Turkey		
*H. glycines*	Hg01	Langfang, Hebei, China	Soybean	[23]
	Hg02	Heilongjiang, China	Soybean	
	Hg03	Henan, China	Soybean	
*H. sojae*	Hso	Jiangxi China	Soybean	[29]
*H. cruciferae*	Hc	Hubei, China	Oilseed rape	This study
*H. avenae*	Ha	Baxing, Beijing, China	Wheat	[30]
*H. filipjevi*	Hf	Xuchang, Henan, China	Wheat	[31]
*H. latipons*	Hl	Belgium ^a^	Wheat	[32]
*H. elachista*	He	Hunan, China	Rice	[33]
*H. amaranthusiae n.* sp	Ham	Xuanwei, Yunnan, China	*Amaranthus retroflexus*	[34]
*H. zeae*	Hz	Henan, China	Maize	[35]
*Globodera rostochiensis*	Gr	Belgium ^a^	Potato	[36]
*G. pallida*	Gp	Belgium ^a^	Potato	
*Cactodera cacti*	Cc	Liaoning, China	Weeds	[37]
*Meloidogyne enterolobii*	Me	Wenchang, Hainan, China	Tomato	[38]
*M. graminicola*	Mg	TikeGyi, Yangon, Myanmar	Rice	[39]
*M. incognita*	Mi	Langfang Hebei, China	Tomato	[38]
*M. hapla*	Mh	Beijing, China	Rose	[38]
*Ditylenchus Destructor*	Dd	Hebei, China	Sweet potato	[40]

^a^ These populations were provided by Prof. Maurice Mones (Institute for Agriculture and Fisheries Research (ILVO), Belgium).

**Table 2:** Codes and sequences of primers used in this study.

Primer Name	Sequence 5′-3′	Types	Production Sizes	References
OPA-01	5′-CAGGCCCTTC-3′	RAPD primers	Random, uncertain	[9,23,41]
OPA-02	5′-TGCCGAGCTG-3′			
OPA-03	5′-AGTCAGCCAC-3′			
OPA-04	5′-AATCGGGCTG-3′			
OPA-05	5′-AGGGGTCTTG-3′			
OPA-06	5′-GGTCCCTGAC-3′			
OPA-09	5′-GGGTAACGCC-3′			
OPA-13	5′-CAGCACCCAC-3′			
OPA-18	5′-AGGTGACCGT-3′			
OPB-15	5′-GGAGGGTGTT-3′			
OPC-06	5′-AAGACCCCTC-3′			
OPD-13	5′-GGGGTGACGA-3′			
OPG-06	5′-GTGCCTAACC-3′			
OPG-08	5′-TCACGTCCAC-3′			
OPK-16	5′-GAGCGTCGAA-3′			
D2A	5′-ACAAGTACCGTGAGGGAAAGTTG-3′	28S-rDNA universal primers	780 bp	[16]
D3B	5′-TCGGAAGGAACCAGCTACTA-3′			
TW81	5’-GTT TCC GTA GGT GAA CCT GC-3′	rDNA-ITS universal primers	1027–1045 bp	[42]
AB28	5’-ATA TGC TTA AGT TCA GCG GGT-3′			
SHF6	5′-GTTCTTACGTTACTTCCA-3′	Specific primer	255 bp	[12]
rDNA2	5′-TTTCACTCGCCGTTACTAAGG-3′	rDNA-ITS universal primer		
OPA06-HsF	5′-GGACCCTGACGACCAGAATA-3′	SCAR primers	922 bp	This study
OPA06-HsR	5′-GACAACACGAAGGAGCGAGC-3′			

**Table 3:** Group treatments of *H. schachtii* with other related species.

Treatments	Mixed Populations	Detection Results
Group 1	*H. schachtii, H. glycines*, *H. sojae*, *H. avenae* and *H. flipjevi*	+
Group 2	*H. schachtii, G. rostochiensis* and *G. pallida*	+
Group 3	*H. schachtii, M. enterolobii*, *M. incognita*, *M. hapla* and *M. graminicola*	+
Group 4	*H. schachtii, D. ditylenchus*	+
Group 5	*H. glycines*, *H. avenae*, *H. flipjevi*, *G. pallida* and *G. rostochiensis*	−
Group 7	*M. enterolobii*, *M. incognita*, *M. hapla* and *M. graminicola*	−
Group 8	*D. ditylenchus*	−
Positive Control	*H. schachtii*	+

“+”, Positive results; “−”, Negative results.

**Table 4:** Detection of *Heterodera schachtii* in naturally infested field samples.

Samples	Host	Location	GPS	Nematode Density in Root ^a^	Cyst Density ^b^	Detection Results		
						SCAR-PCR	PCR	ITS-Sequencing
1	Sugar beet	Huocheng County-1, Xinjiang	N44°4′7″, E80°44′36″	0	0	-	-	N/A
2		Huocheng County-2, Xinjiang	N44°2′43″, E80°50′7″	0	0	-	-	N/A
3		Qapqal County-1, Xinjiang	N43°45′50″, E80°59′34″	0	4	-	-	*H. avenae*
4		Qapqal County-2, Xinjiang	N43°46′26″, E81°2′6″	0	0	-	-	N/A
5		Qapqal County-3, Xinjiang	N43°45′40″, E81°9′18″	0	0	-	-	N/A
6		Xinyuan County-1, Xinjian	N43°27′09″, E 82°59′56″	22 ± 7	132	+	+	*H. schachtii*
7		Xinyuan County-2, Xinjian	N43°29′55”, E83°59′25”	44 ± 6	144	+	+	*H. schachtii*
8		Xinyuan County-3, Xinjian	N43°44′67”, E83°06′39”	12 ± 3	287	+	+	*H. schachtii*
9		Xinyuan County-4, Xinjian	N43°31′04”, E83°11′13”	23 ± 7	92	+	+	*H. schachtii*
10		Xinyuan County-5, Xinjian	N42°88′3252”, E83°39′710”	33 ± 5	54	+	+	*H. schachtii*
11		Emin County-1, Xinjiang	N46°46′460”, E83°53′902”	8 ± 3	11	+	+	*H. schachtii*
12		Emin County-2, Xinjiang	N46°46′468”, E83°53′949”	45 ± 11	64	+	+	*H. schachtii*
13		Keyouqian Banner, Inner Mongolia	N/A	0	3	-	-	*H. avenae*
14		Urad Front Banner, Inner Mongolia	N/A	0	12	-	-	*H. glycines*
15		Linxi County, Inner Mongolia	N/A	0	9	-	-	*H. avenae* *H. glycines*

^a^ Numbers of *Heterodera* spp. counted after staining with acid fuchsin in 0.1 g sugar beet. ^b^ Numbers of cyst isolated and counted from 100 mL soil. N/A, no record.

## Data Availability

The original contributions presented in the study are included in the article. Further inquiries can be directed to the corresponding author/s.

## Supplementary Materials

The following are available online at https://www.mdpi.com/article/10.3390/life11121358/s1, Figure S1: sequences alignment between *H. schachtii* and other homologous nematodes. HS-OPA06: *H. schachtii* (GenBank accession No. MW854319); Hsc_scaff211: *H. schachtii* (JAHGVF010000211.1); Hg_X12_chr4: *H. glycines* (VAPQ01000257.1); Gr_Scaffold36: *G. rostochiensis* (JAEVLO010000036.1); Gpal_scaffold_689_contig_3: *G. pallida* (CBXT010012390.1); Ge_scaf_0021: *G. ellingtonae* (MEIZ01000021.1); Mi_W1_scaffold8403_cov70: *M. incognita* (RCFL01002996.1); Ma_A2-0_rig00001048: *M. arenaria* (RCFL01000363.1); Mj_VW4_scaffold15760_cov276: *M. javanica* (RCFK01013271.1); Me_Ment3s03051: *M. enterolobii* (CAJEWN010003051.1). Figure S2: PCR amplification products from inoculated roots and soil samples using published PCR assay with SHF6 and rDNA2 primers.
