# Supplementary figures and images for: Development of a Species-Specific SCAR-PCR Assay for Direct Detection of Sugar Beet Cyst Nematode (Heterodera schachtii) from Infected Roots and Soil Samples
Source: Life (Basel). 2021 Dec 7;11(12):1358. doi: 10.3390/life11121358 (PMC8708203; doi:10.3390/life11121358)

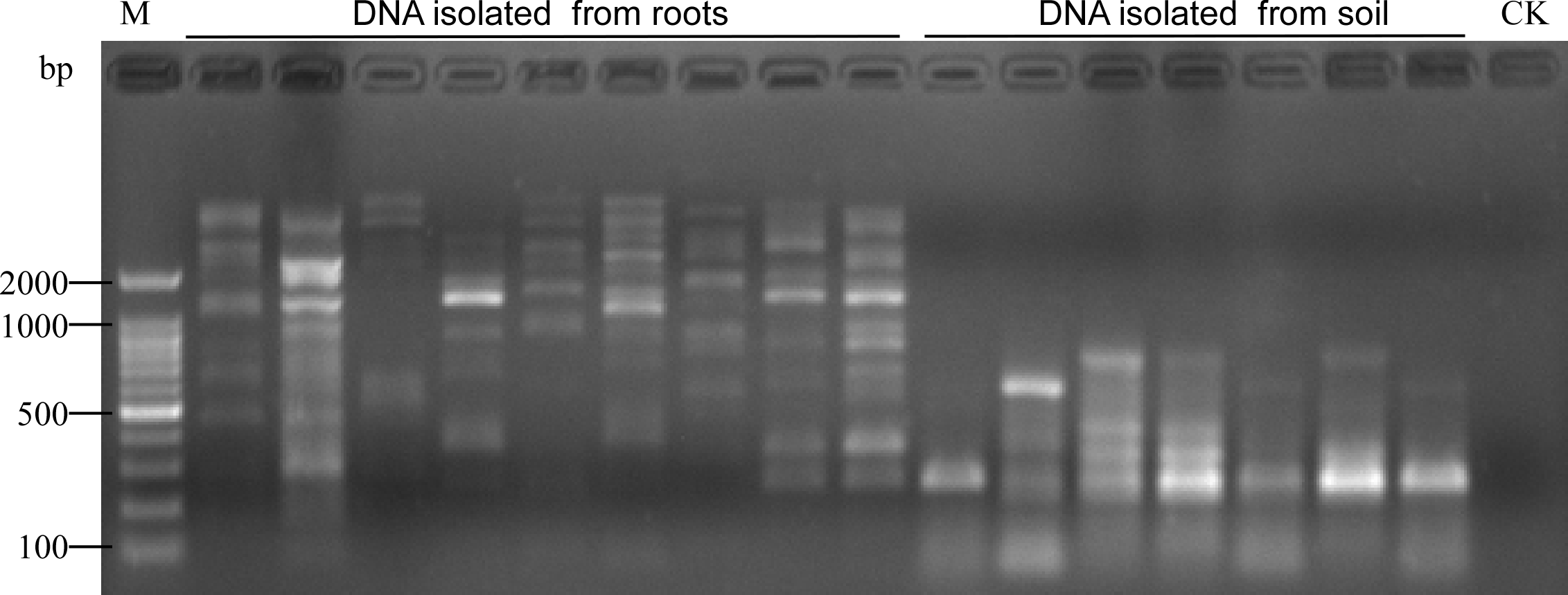

Supplement: Supplementary file 1 [file life-11-01358-s001.zip › Figure S2.tif]

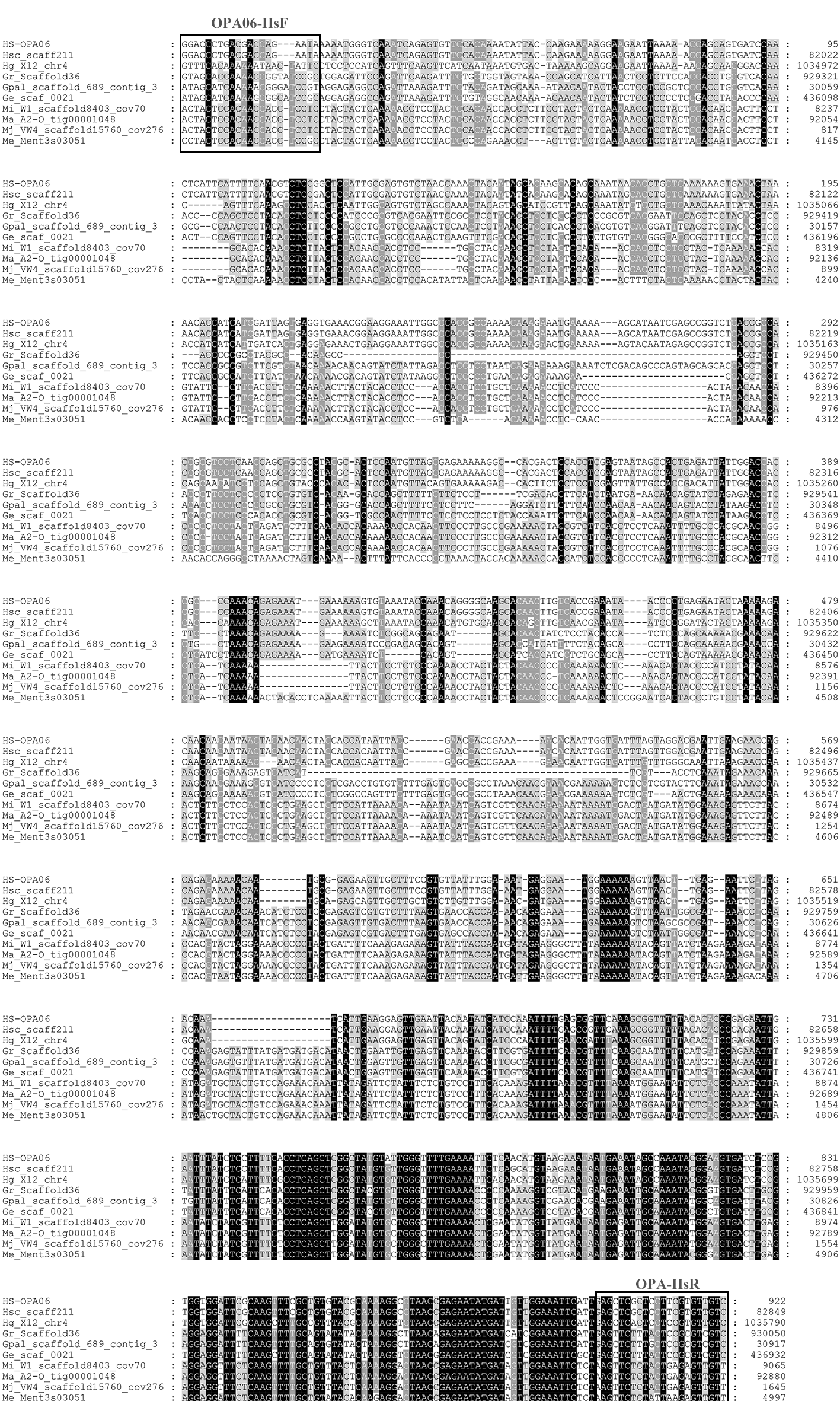

Supplement: Supplementary file 1 [file life-11-01358-s001.zip › Figure S1.tif]
